# Time-dependent effects of ultraviolet and nonthermal atmospheric pressure plasma on the biological activity of titanium

**DOI:** 10.1038/srep33421

**Published:** 2016-09-15

**Authors:** Sung-Hwan Choi, Won-Seok Jeong, Jung-Yul Cha, Jae-Hoon Lee, Hyung-Seog Yu, Eun-Ha Choi, Kwang-Mahn Kim, Chung-Ju Hwang

**Affiliations:** 1 Department of Orthodontics, The Institute of Cranio-Facial Deformity, College of Dentistry, Yonsei University, Seoul, 03722, Korea; 2 Department and Research Institute of Dental Biomaterials and Bioengineering, BK21 PLUS Project, College of Dentistry, Yonsei University, Seoul, 03722, Korea; 3 Department of Prosthodontics, College of Dentistry, Yonsei University, Seoul, 03722, Korea; 4 Department of Electrobiological Physics, Department of Plasma Bioscience Display, Department of Defence Acquisition, Plasma Bioscience Research Center, Kwangwoon University, Seoul, 01897, Korea

## Abstract

Here, we evaluated time-dependent changes in the effects of ultraviolet (UV) and nonthermal atmospheric pressure plasma (NTAPPJ) on the biological activity of titanium compared with that of untreated titanium. Grade IV machined surface titanium discs (12-mm diameter) were used immediately and stored up to 28 days after 15-min UV or 10-min NTAPPJ treatment. Changes of surface characteristics over time were evaluated using scanning electron microscopy, surface profiling, contact angle analysis, X-ray photoelectron spectroscopy, and surface zeta-potential. Changes in biological activity over time were as determined by analysing bovine serum albumin adsorption, MC3T3-E1 early adhesion and morphometry, and alkaline phosphatase (ALP) activity between groups. We found no differences in the effects of treatment on titanium between UV or NTAPPJ over time; both treatments resulted in changes from negatively charged hydrophobic (bioinert) to positively charged hydrophilic (bioactive) surfaces, allowing enhancement of albumin adsorption, osteoblastic cell attachment, and cytoskeleton development. Although this effect may not be prolonged for promotion of cell adhesion until 4 weeks, the effects were sufficient to maintain ALP activity after 7 days of incubation. This positive effect of UV and NTAPPJ treatment can enhance the biological activity of titanium over time.

Titanium is commonly used in prosthetic implants for restoring joint function and relieving pain in joint arthroplastic operation, in dental implants for rehabilitation of missing teeth, and as an absolute skeletal anchorage, because the oxidised titanium surface exhibits excellent biological compatibility and can achieve tight mutual contact with adjacent bone without formation of fibrous tissue surrounding the implants, a feature called osseointegration^1^. Nevertheless, the rate of revision surgery for orthopaedic joint implants is over 10% within 15 years of the initial surgery, primarily owing to aseptic loosening through lack of sufficient bone-implant integration without concurrent trauma or infection^2^,^3^. Five-year success rates for titanium dental implants range from 90.1% to 96.5% for the fixed prosthesis type; however, the success rates decrease over time, reaching 89% and 83% after 10 and 16 years, respectively^4^. Patients at higher risk, i.e. those with bone compromised by systemic diseases, aging, or previous periodontal disease, exhibit higher long-term failure rates^5^,^6^. Such implant failure can lead to increased patient dissatisfaction and high socioeconomic burden, particularly in older patients.

In order to prevent or reduce the possibility of implant failure, various topographical modifications to the titanium surface, such as sand-blasted, large grit, acid etched (SLA) or anodic oxidation, have been used to increase surface roughness and thereby improve surrounding osteoblastic cell adhesion, proliferation, and differentiation^7^,^8^,^9^,^10^. However, previous studies have reported that these surface modifications are limited to activation of the bioinert titanium surface because the bioactivity and osteoconductivity of the titanium surface decrease over time and because commercially available titanium devices are sold as sufficiently aged with packaging, regardless of the type of surface treatment^11^,^12^,^13^.

Recently, ultraviolet (UV) or nonthermal atmospheric pressure plasma jet (NTAPPJ) treatment has been shown to modify the physicochemical properties of titanium and to enhance its biologic capability without altering topography^13^,^14^,^15^,^16^,^17^. These treatments can change the titanium surface from hydrophobic to hydrophilic due to removal of surface hydrocarbon and/or formation of chemically reactive hydroxyl radical species with reduced surface negative charge^18^,^19^,^20^. Moreover, Bacakova *et al.* reported that cell adhesion was promoted by a moderately hydrophilic and less negatively charged surface^21^. However, most previous studies have only investigated cellular responses immediately after treatment by each method. For example, Canullo *et al.* reported that the beneficial effects of various titanium implanted surfaces immediately after argon plasma treatment for 12 min were comparable to those immediately after UV treatment for 3 h *in vitro*^22^. Based on the potential for clinical application, the study included considerably different irradiation times for the two methods, although the UV irradiation time could probably be shortened using higher UV flux lamps. Additionally, the study focused only on the immediate effects of treatment on the cellular response.

To the best our knowledge, few studies have evaluated the effects of treatment on the titanium surface between UV and NTAPPJ or time-dependent aging of the titanium surface after UV or NTAPPJ. Each method has been successfully applied with increased bone-implant contact *in vivo*, and no significant intergroup differences in histological inflammatory reactions by the recipient’s immune system have been identified^23^,^24^,^25^,^26^. However, in order to ensure the validity of UV or NTAPPJ treatment before clinical application, it is necessary to confirm that treatment effects are maintained for at least up to 4–8 weeks, during the early healing time for bone formation after implantation^27^,^28^.

Therefore, in this study, we aimed to evaluate time-dependent changes in the effects of UV and NTAPPJ on the biological activity of titanium.

## Materials and Methods

### Preparation of titanium samples

Titanium samples were prepared in a disc shape (12.0 mm in diameter, 1.0 mm thickness) by machining commercially of pure titanium (grade IV; Osstem Implant Co., Seoul, Korea). The titanium discs were sequentially cleaned with acetone, alcohol, and distilled water for 15 min each using an ultrasonic cleaner and then sterilised using ethylene oxide (EO) gas at a temperature of 55 °C for 1 h^16^,^29^.

The prepared titanium discs were stored in sealed 12-well cell culture plates under dark ambient conditions at room temperature over 8 weeks for a full aging^14^,^30^. After the storage, some titanium discs were treated by UV or NTAPPJ for a similar time. UV light irradiation was carried out for 15 min using a photo device (TheraBeam Affiny; Ushio Inc., Tokyo, Japan). The UV light was delivered as a mixture of spectra via a UV lamp, and the measured intensities were 0.05 mW/cm^2^ (λ = 360 ± 20 nm) and 2 mW/cm^2^ (λ = 250 ± 20 nm)^31^. NTAPPJ treatment was performed with a compressed air gas flow of 5000 sccm for 10 min using a device manufactured by the Plasma Bioscience Research Center (Kwangwoon University, Seoul, Korea)^32^. Briefly, the distance between the plasma jet tip and the titanium sample surface was set to 3 mm, and the maximum voltage was set to 17 kV. This NTAPPJ device consisted of a stainless steel inner electrode with 1.2 mm depth and 0.2 mm thickness along with quartz (3.2 mm depth) as the dielectric. These UV- or NTAPPJ-treated titanium discs were used immediately for each experiment or stored under dark ambient conditions for 3, 7, 14, or 28 days before starting each experiment. The control group was defined as sufficiently aged titanium discs without any treatment.

### Surface characterisation

The surface morphologies of the samples were examined immediately after treatment by UV or NTAPPJ and for the untreated control group using scanning electron microscopy (SEM; Hitachi S3000N; Hitachi, Tokyo, Japan) and an optical three-dimensional surface profiler (ContourGT; Bruker, AZ, USA) using the vertical scanning interferometry (VSI) mode with a green luminous source. Surface roughness parameters, including average roughness (Sa) and peak-to-valley roughness (Sz) values, were measured at a magnification of 10× with a scanning area of 310 μm × 230 μm.

Changes in the hydrophilicity of the titanium disc surface after treatment over time were assessed by measuring the contact angle and spread area of a 4-μL H_2_O droplet on the centre of each sample surface. Ten seconds after the drop fell on the surface, the data were captured using a video contact angle goniometer (Phoenix 300; SEO, Gyeonggi-do, Korea) to calculate the contact angle and spread area using Image XP software (SEO) immediately or at 3, 7, 14, or 28 days after UV or NTAPPJ treatment.

The chemical composition of the titanium disc surface immediately and 28 days after treatment using UV or NTAPPJ was evaluated using X-ray photoelectron spectroscopy (XPS; K-alpha; Thermo VG, UK), operated using a monochromatic Al Ka line (1486.6 eV) with the following parameters: 12 kV, 3 mA, and a spot size of 400 μm. The titanium, oxygen, and carbon contents were examined under vacuum conditions at each time point.

### Zeta potential

To investigate changes in the zeta potential of the titanium disc surface immediately and 28 days after treatment using UV or NTAPPJ, the samples were dispersed with monitor particles (polystyrene latex) in a high-purity silica glass cell. This glass cell was connected into a laser electrophoresis spectroscope (ELSZ 1000; Otsuka Electronics Co., Osaka, Japan) to measure the zeta potential of the surface^33^. The measurements were performed in 10 mM NaCl solution at pH 7.4. The data were selected when the distribution of zeta potential according to the height of the cuvette was parabolic from the centre. The electrokinetic streaming potential was automatically calculated using the Smoluchowski method.

### Protein adsorption assay

Bovine serum albumin, fraction V (BSA; Pierce Biotechnology, Inc., IL, USA) was used as a model protein. The protein solution (100 μM; 1 mg/mL in phosphate-buffered saline [PBS], pH 7.4) was pipetted onto and spread over each sample surface immediately and 28 days after treatment using UV or NTAPPJ treatment. After 4 h of incubation under sterile humidified conditions at 37 °C in 5% CO_2_, nonadherent protein removed, and the initial whole solution was mixed with microbicinchoninic acid (Pierce Biotechnology, Inc., IL, USA) in new 96-well cell culture plates, followed by incubation at 37 °C for 30 min. The optical density (OD) of each sample was quantified using a microplate reader (Epoch, BioTek Instruments, VT, USA) at 562 nm, and the rate of protein adsorption was calculated as the percentage of albumin adsorbed to the sample surface relative to the total amount using a BSA standard curve provided with the kit.

### Cell culture

Murine MC3T3-E1 osteoblast cells (CRL-2593; American Type Culture Collection, VA, USA) were used at passages 7–9, regardless of storage time, to determine the cellular responses to the treatments. The cells were cultured in alpha-MEM cell culture medium (Gibco, NY, USA) containing 10% foetal bovine serum (FBS; Gibco), penicillin (100 U/mL; Gibco), and streptomycin (100 mg/mL; Gibco) at 37 °C in 5% CO_2_. After reaching 80% confluence, the cells were detached using 0.25% trypsin/1 mM EDTA-4Na (Gibco) to prevent contact inhibition. The cell culture medium was changed every 48 h.

### Cell adhesion assay

A total of 1 × 10^4^ cells in 100 μL was placed onto each sample surface in a 24-well plate immediately and 28 days after treatment with UV or NTAPPJ. After 4 or 24 h of incubation, these quantifications were performed using water-soluble tetrazolium salt (WST)-based colorimetry (EZ-1000; DoGenBio Co., Gyeonggi-do, Korea). The cells were incubated at 37 °C for 4 h with tetrazolium salt (WST) reagent, and the amount of formazan product was measured using a microplate reader (Epoch; BioTek Instruments) at 450 nm. The results were expressed as the relative percentage of cells attached to the sample surface compared with that of the control group.

### Cell morphology and morphometry

After incubation of cells on treated or untreated titanium disc surfaces for 4 h at 37 °C in 5% CO_2_, cells were stained using diamidino-2-phenylindole, dihydrochloride (DAPI; blue for nuclei; Molecular Probes, Invitrogen, NY, USA) and rhodamine phalloidin (red for F-actin filaments; Molecular Probes). Confocal laser-scanning microscopy (LSM 700; Carl Zeiss, Jena, Germany) was used to examine cell morphology and cytoskeletal arrangement. Quantitative assessment of cell area, perimeter, and Feret’s diameter was performed using ImageJ software (NIH, Bethesda, MD, USA).

### Alkaline phosphatase (ALP) activity

After incubation of cells on treated or untreated titanium discs for 7 days, cells were lysed with 0.2% Triton X-100 (Sigma-Aldrich, Inc., MO, USA). The lysates were then centrifuged, and the supernatants were reacted with *p*-nitrophenylphosphate (*p*NPP) substrate from an ALP assay kit (SensoLyte *p*NPP Alkaline Phosphatase Assay Kit; AnaSpec, CA, USA) at room temperature for 60 min. The optical density (OD) was read at 405 nm using a plate reader (Epoch; BioTek Instruments).

### Statistical analysis

All statistical analyses were performed using IBM SPSS software, version 21.0 (IBM Korea Inc., Seoul, Korea) for Windows. According to previous studies^16^,^20^,^22^, at least four samples for each experiment were used, and each experiment was repeated three times. The results between three groups (the control, UV, and NTAPPJ) at each time point were analysed by one-way analysis of variance (ANOVA) with Tukey’s method. Differences with *P* values of less than 0.05 were considered statistically significant.

## Results

### Surface characterisation

**Figure 1:**
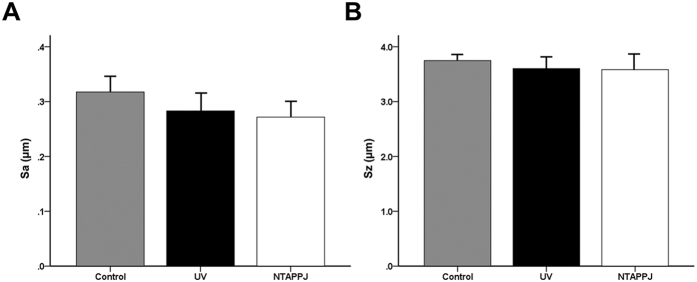
Results of three-dimensional (3D) surface topographic analysis of the titanium disc surface immediately after UV and NTAPPJ compared with that of the control group. Surface roughness parameters, Sa (**A**) and Sz (**B**), were quantitatively measured at a magnification of 10× with a scanning area of 310 μm × 230 μm, and the results were compared between groups.

SEM analysis confirmed that the titanium discs used in this study showed typical lathe marks left by the milling process for machined titanium surfaces. The UV- or NTAPPJ-treated titanium discs showed no marked differences in surface roughness parameters, including Sa and Sz, as compared with the control group under tridimensional analysis (Fig. 1A,B). Sa values of the control, UV-treated, and NTAPPJ-treated groups were 0.32 ± 0.03, 0.28 ± 0.05, and 0.27 ± 0.03 μm, respectively (*P* > 0.05). Sz values of the control, UV-treated, and NTAPPJ-treated groups were 3.75 ± 0.15, 3.60 ± 0.28, and 3.58 ± 0.37 μm, respectively (*P* > 0.05).

**Figure 2:**
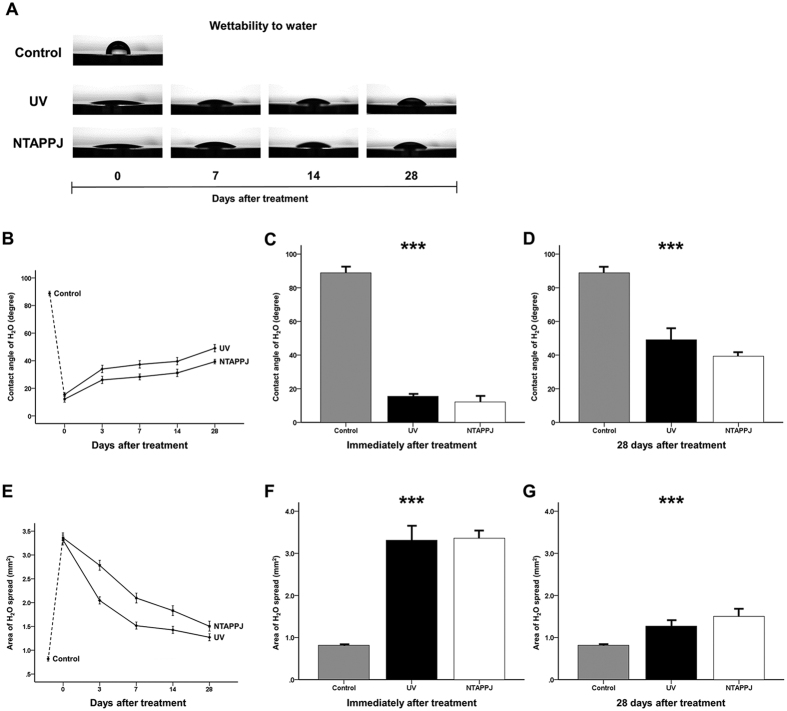
Changes in the hydrophilicity of the titanium disc surface after treatment over time. (**A**) Changes in wettability by water over time, as measured using a 4-μL H_2_O droplet on the centre of each sample surface, between UV- and NTAPPJ-treated discs. (**B**) Changes in the contact angle with the titanium disc surface over time for the UV- and NTAPPJ-treated groups. Comparison of changes in the contact angle immediately (**C**) and 28 days (**D**) after treatment between groups. (**E**) Changes in the spread area of the titanium disc surface over time. Comparison of changes in the spread area immediately (**F**) and 28 days (**G**) after treatment between groups. ***P < 0.001 for comparisons between the indicated groups.

However, there was a significant difference in wettability by water between groups (Fig. 2A–G). As shown in Fig. 2A, the H_2_O droplet did not spread and maintained an arc shape on the titanium disc surface in the control group. The contact angle and spread area of the control group were 89.56 ± 3.97° and 0.82 ± 0.04 mm^2^, respectively. In contrast, in the UV- and NTAPPJ-treated groups, although the hydrophilicity was decreased markedly, with the H_2_O droplet gradually changing into an arc shape over time, the droplet spread extensively over the surface as compared with that in the control group, regardless of the storage time (*P* < 0.001). The contact angles and spread areas on the titanium surface were shifted from 15.50 ± 1.79° to 49.08 ± 7.84° and from 3.31 ± 0.39 mm^2^ to 1.27 ± 0.17 mm^2^, respectively, at 0 and 28 days after UV treatment, respectively. Similarly, the contact angles and spread areas on NTAPPJ-treated titanium discs were also shifted from 12.14 ± 3.14° to 39.31 ± 2.36° and from 3.36 ± 0.20 mm^2^ to 1.50 ± 0.21 mm^2^, respectively, over time. There were no significant differences in contact angles and spread areas between the UV- and NTAPPJ-treated groups, regardless of the storage time.

### Changes in surface chemical compositions in the UV- and NTAPPJ-treated groups

**Figure 3:**
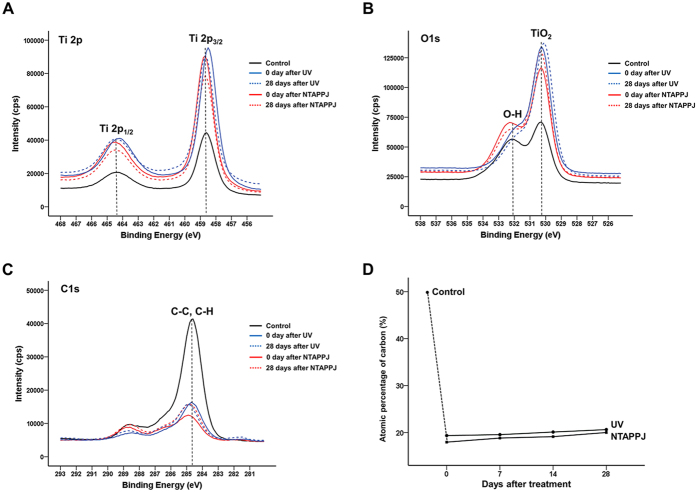
Changes in the chemical composition of the titanium disc surface after treatment over time. Changes in Ti2p (**A**), O1s (**B**), and C1s (**C**) spectra immediately and 28 days after treatment between groups. (**D**) Changes in atomic percentages of carbon over time after treatment between groups.

As shown in Fig. 3A, the peaks corresponding to the Ti2p 1/2 and Ti2p 3/2 components were located at binding energies from 458.7 to 464.3 eV, and the peaks in the experimental groups were higher than those in the control group, regardless of the storage time. The major peak corresponding to TiO_2_ of the O1s spectra was located at a binding energy of 530.1 eV and was increased in the experimental groups compared with that in the control group, regardless of the storage time (Fig. 3B). In particular, the peak corresponding to the hydroxyl group (-OH) at a binding energy of 532.0 eV for the NTAPPJ-treated titanium discs was increased compared with those of UV-treated and control titanium discs. However, the peaks of both UV- and NTAPJJ-treated groups were markedly decreased over time. In the C1s peaks, the peak corresponding to the hydrocarbon (-CH) at a binding energy of 284.7 eV was decreased in the experimental groups compared with that in the control group (Fig. 3C). Similarly, the atomic percentages of carbon in the UV- and NTAPPJ-treated groups were lower than those in the control group, regardless of the storage time. The carbon contents immediately after UV or NTAPPJ treatment were markedly shifted from 49.48% to 17.95% or 19.35%, respectively, and increased slightly to about 20% at 28 days after treatment (Fig. 3D).

### Decreased negative charges in UV- and NTAPPJ-treated groups

**Figure 4:**
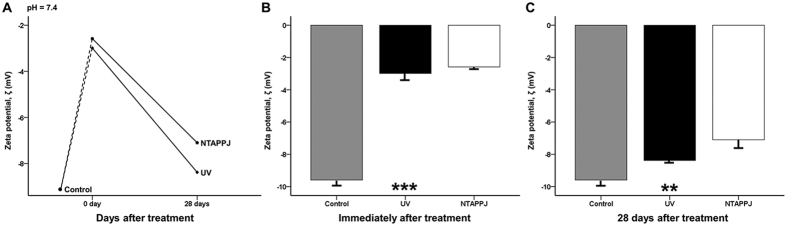
(**A**) Changes in the zeta potential of the titanium disc surface after treatment over time at pH 7.4. Comparison of changes in zeta potential immediately (**B**) and 28 days (**C**) after treatment between groups. **P < 0.01, ***P < 0.001 for comparisons between the indicated groups.

The zeta potential of the sample surface of the control group was highly negative (−9.59 ± 0.33 mV) at pH 7.4 (Fig. 4A–C). The zeta potentials increased immediately after UV or NTAPPJ treatment to −2.99 ± 0.43 and −2.58 ± 0.12 mV, respectively, and were significantly different compared with those of the control group (*P* < 0.001; Fig. 4B). At 28 days after treatment, the zeta potentials of UV- and NTAPPJ-treated groups were markedly decreased to −8.38 ± 0.17 and −7.10 ± 0.53 mV, respectively, and were significantly different compared with those of the control group (*P* = 0.001; Fig. 4C). There were no significant differences in the zeta potentials of the UV- and NTAPPJ-treated groups, regardless of the storage time.

### Protein adhesion capacity in the UV- and NTAPPJ-treated groups

**Figure 5:**
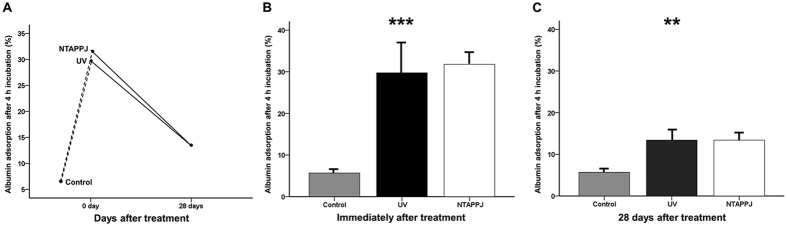
(**A**) Changes in albumin adsorption rates on the titanium disc surface after treatment over time. Comparison of changes in albumin adsorption rates immediately (**B**) and 28 days (**C**) after treatment between groups. **P < 0.01, ***P < 0.001 for comparisons between the indicated groups.

Immediately after UV or NTAPPJ treatment, the amounts of BSA adsorbed to the titanium surface during the 4-h experimental period were significantly greater than those of the control group (*P* < 0.001; Fig. 5A). The rate of BSA adsorption to the titanium discs relative to the total protein in the control group was 5.68% ± 1.06%. In UV- and NTAPPJ-treated groups immediately after treatment, the rates of BSA adhesion to the titanium discs increased to 29.73% ± 8.64% and 31.78% ± 2.72%, respectively (Fig. 5B). These rates decreased to about 13.4% (UV, 13.40% ± 3.06%; NTAPPJ, 13.40% ± 1.89%) at 28 days after treatment. Although these rates were not different between experimental groups, significant differences were observed compared with the control group, indicating that the electrical polarity of the 28-day-old treated titanium disc surface was sufficient for induction of albumin adhesion to the titanium surface compared with that of the control group (*P* = 0.004; Fig. 5C).

### Cellular adhesion capacity in UV- and NTAPPJ-treated groups

**Figure 6:**
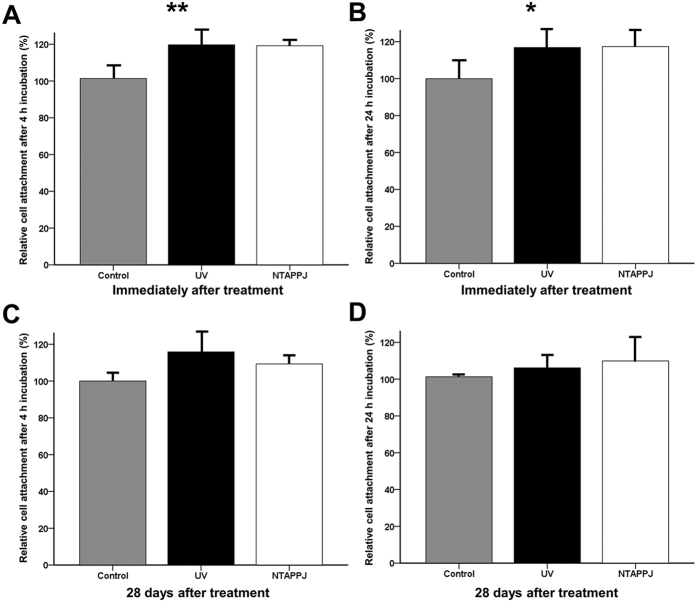
Changes in relative osteoblastic cell attachment rates on the titanium disc surface after treatment over time. Cell attachment rates immediately after treatment after 4 h (**A**) and 24 h (**B**) of incubation and 28 days after treatment after 4 h (**C**) and 24 h (**D**) of incubation. *P < 0.05, **P < 0.01 for comparisons between the indicated groups.

**Figure 7:**
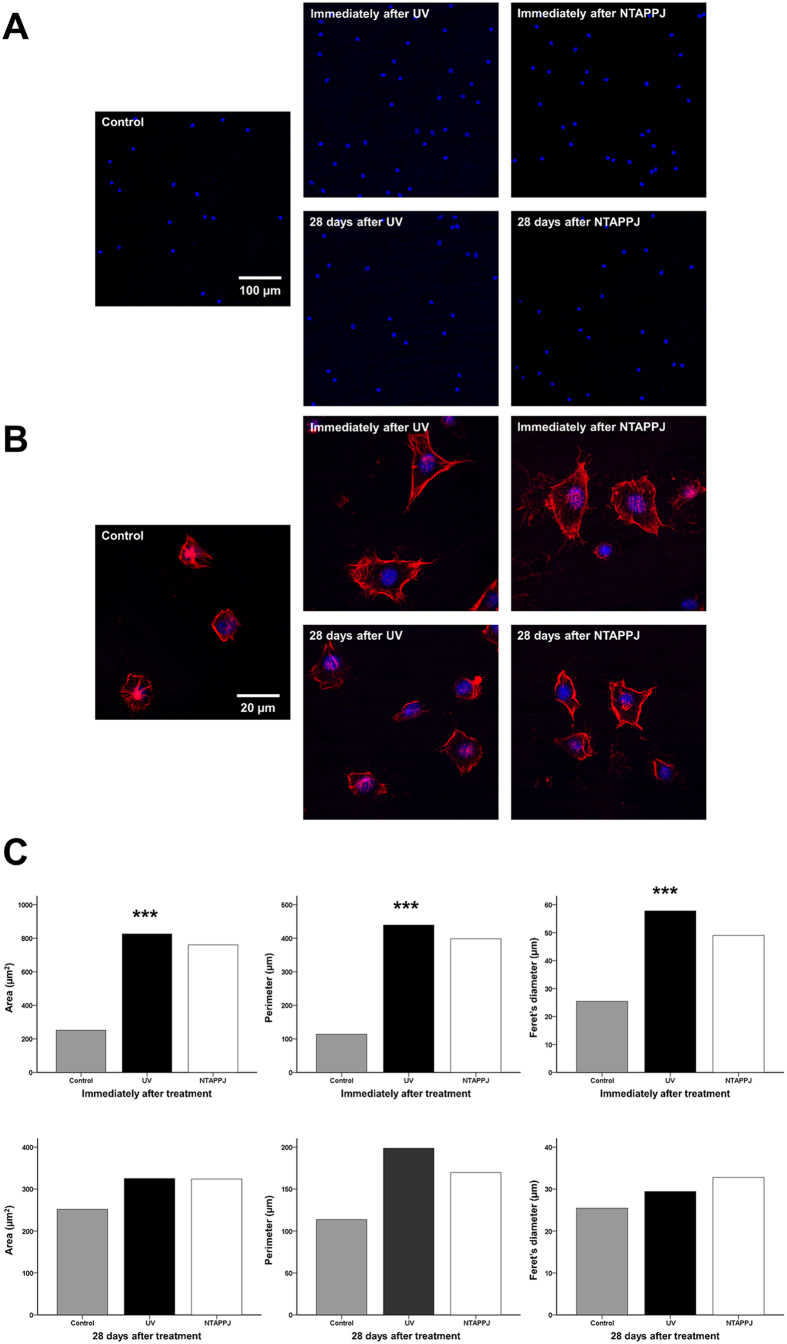
Changes in cellular morphology on the titanium disc surface after treatment over time. Fluorescent stained (**A**) blue coloration for nuclei using DAPI and (**B**) red coloration for F-actin filaments using rhodamine phalloidin. (**C**) Comparison of cytoskeleton development, including area, perimeter, and Feret’s diameter of the cells, after treatment at each time point. ***P < 0.001 for comparisons between the indicated groups.

After 4 or 24 h of incubation, the number of adherent cells was increased for UV- and NTAPPJ-treated samples used immediately after treatment as compared with that of the control group (Fig. 6A,B). The greater number of attached cells was observed on the titanium disc surface immediately after treatment using UV or NTAPPJ compared with that of the control group and 28-day-old treated titanium discs (Fig. 7A). When the cellular attachment ratio of the control group was set at 100%, the relative cellular attachment ratios on UV- or NTAPPJ-treated surfaces were significantly increased to about 119% (UV, 119.69% ± 8.87%; NTAPPJ, 119.18% ± 3.39%) after incubation for 4 h (*P* = 0.002; Fig. 6A) and about 117% (UV, 116.86% ± 10.68%; NTAPPJ, 117.27% ± 7.88%) after incubation for 24 h (*P* = 0.049; Fig. 6B) as compared with that in the control group. However, 28 days after treatment, there were no significant differences in cellular attachment between groups, regardless of the incubation time, indicating that the 28-day-old treated titanium disc surface was not able to promote osteoblastic cell adhesion to the titanium disc surface, regardless of the type of treatment (Figs 6C,D and 7A).

### Changes in cellular morphology in the UV- and NTAPPJ-treated groups

After 4 h of incubation, larger osteoblastic cells with extended actin filaments and a spindle shape were observed on UV- and NTAPPJ-treated titanium discs used immediately after treatment as compared with that in the control group, which exhibited a circular shape (Fig. 7B). The mean cell area, perimeter, and Feret’s diameter of the osteoblastic cells on the titanium disc surface immediately after UV or NTAPPJ treatment were significantly greater than those of the control group (*P* < 0.001; Fig. 7C). However, 28 days after treatment, there were no marked differences in cytomorphology between the three groups, indicating that there was a significantly delay in cellular spread and cytoskeleton development when cells were grown on the surfaces of 28-day-old treated titanium discs (Fig. 7B,C).

### Enhanced ALP activity in UV- and NTAPPJ-treated groups

**Figure 8:**
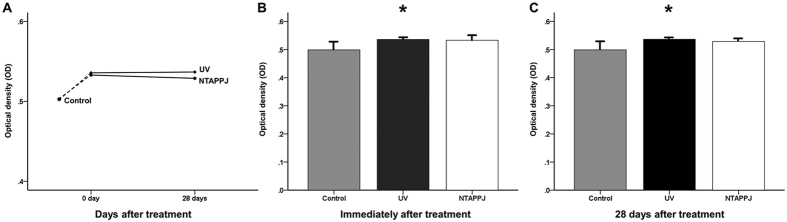
(**A**) Changes in ALP activity of the titanium disc surface after treatment over time, as determined by measuring the optical density (OD). Comparison of OD values immediately (**B**) and 28 days (**C**) after treatment between groups. *P < 0.05 for comparisons between the indicated groups.

After 7 days of incubation, the ODs of UV- and NTAPPJ-treated titanium discs were slightly but significantly greater than that of the control group, regardless of the storage time (Fig. 8A–C; *P* = 0.015 at immediately after treatment and *P* = 0.011 at 28 days after treatment). The ALP activity of the experimental groups was relatively constant over time, indicating that the numbers of living cells on the treated titanium disc surfaces were greater than that of the control group, regardless of the storage time, after 7 days of incubation.

## Discussion

In this study, we aimed to evaluate time-dependent changes in the effects of UV and NTAPPJ on the biological activity of titanium. Importantly, we found that there were no differences in surface characteristics, protein adsorption, and cellular responses between UV and NTAPPJ treatments regardless of the storage time. The effects of 28-day-old treated titanium discs were not sufficient to enhance cell attachment and cytoskeleton development compared with the control group. However, ALP levels, indicating the degree of cellular differentiation, were maintained, regardless of the type of treatment and storage time. These data provide important insights into the effects of surface modifications on titanium implants for clinical applications.

The physical and reactive chemistry of NTAPPJ can be derived from the production of an electric field capable of ionising air or a carrier gas, such as nitrogen, helium, and argon, at atmospheric pressure^34^,^35^. From economic and clinical perspectives, it may also be desirable to utilise gases that are less expensive and easily available in the clinic, such as air, for applications involving NTAPPJ. Seo *et al.* also reported that air-based NTAPPJ using clinical-grade compressed air for 10 min was sufficient to increase cellular responses on the titanium nanotube surface as most dental clinics have built-in air compressors^16^. Based on the above-mentioned information, this study selected air-based NTAPPJ for 10 min to treat the titanium discs.

In this study, UV and NTAPPJ treatment did not significantly alter the surface roughness parameters when analysed immediately after treatment; however, both methods increased the hydrophilicity and wettability of the titanium disc surface. Aita *et al.* reported that UV treatment decreases the percentage of hydrocarbons on the titanium surface without any changes to the surface roughness. Additionally, these physicochemical changes are associated with the photocatalytic phenomena of TiO_2_, and the hydrocarbon level is strongly associated with the rates of protein adsorption and cell attachment^13^. Similarly, NTAPPJ causes an increase in hydrophilicity and a decrease in contact angle due to the effects of removal of hydrocarbon from the titanium surface^15^,^16^,^18^,^36^.

Nevertheless, most previous studies investigating the effects of UV and NTAPPJ on hydrophilicity did not consider the duration of the effect or the consequent rehydrophobisation (i.e. decreased hydrophilicity) of the titanium surface after such treatment, which occurs rapidly in air^30^,^37^,^38^. Our results showed that the hydrophilicity of the titanium disc surface decreased rapidly within 3 days in both the UV- and NTAPPJ-treated groups, reaching half that of the control group by 28 days after treatment. Consistent with the above results, XPS showed that the peak corresponding to C1s and the atomic percentage of carbon increased over time. The increase in carbon content in the experimental groups was small; however, its effects may have a great impact on changes in hydrophilicity and on cellular responses because the hydrophobic hydrocarbon-contaminated surface can cause entrapment of air bubbles, interfering with the interaction between proteins and cells^37^.

We found that the peak corresponding to the hydroxyl groups (-OH) of UV- and NTAPPJ-treated titanium discs were increased compared with those in the control group. During UV treatment, when removing the hydrocarbons from the TiO_2_ surface, photolysis creates an electron-hole pair because of electrons in the valence band of the semiconductor, which transition to the conduction band^20^,^39^. This phenomenon causes the generation of surface oxygen vacancies and reactive oxygen species (ROS), such as the hydroxyl radical.

In particular, NTAPPJ treatment caused an increase in ROS on the titanium surface, as measured immediately after treatment, compared with that in the UV-treated and control groups; consequently, the level of hydrophilicity and surface zeta potential of the NTAPPJ-treated titanium surface were relatively higher than those of the UV-treated titanium surface. The resulting products, such as energetic ions, UV/vacuum UV radiation, charged particles, and ROS, have broadened the scope of NTAPPJ for medical applications from sterilisation to cancer treatment^40^,^41^,^42^,^43^. The distribution of hydroxyl radicals generated from the plasma plume is affected by the presence of a target, its nature (electrical conductivity) and humidity (dry and wet surface), the gas flow rate, and the voltage amplitude^44^,^45^,^46^. Norberg *et al.* revealed that the surface charge of metal is virtually instantly dissipated and the electric field produced at the surface of the metal substrate is created by the accumulation of positively charged species near the surface as the electrons flow into the metal^42^.

In this study, immediately after treatment, the surface zeta potential and albumin adsorption capacity were significantly increased. In contrast, in 28-day-old treated titanium discs, these effects were decreased but were still higher than those in the control group. Untreated titanium surface that have aged for a sufficient period are known to be electronegatively charged, similar to serum albumin molecules^12^. After treatment using UV or NTAPPJ, the negative charge on the titanium surface decreased and consequently increased the protein adsorption rates. These chemo-attractions can enhance cell adhesion ratios because extracellular matrix protein also has a negative charge. In this study, in discs used immediately after UV or NTAPPJ treatment, the amount of cell attachment increased after 4 and 24 h of incubation. In addition, the rates of cell attachment tended to plateau after 4 h of incubation, indicating that the process of cell attachment was accelerated by these treatments. However, 28-day-old treated titanium discs did not exhibit increased cell adhesion compared with the control group. Cellular morphometry also showed that the level of cytoskeleton development on the surface of 28-day-old treated titanium discs was not significantly different between groups. However, ALP levels, indicating the degree of early cellular differentiation and the amount of living cells on the treated titanium disc surface, were maintained, regardless of the type of treatment and storage time.

As mentioned above, these results revealed that the effects of treatment using UV or NTAPPJ on the titanium surface, which altered the negatively charged, hydrophobic (bioinert) surface to a relatively positively charged, hydrophilic (bioactive) surface, may not last to promote surrounding cell adhesion 4 weeks after treatment. However, this effect could still enhance osteoblast maturation after 7 days of incubation compared with that in the control group, which exhibited a hydrophobic surface.

When placing titanium implants, the first healing step is formation of a fibrin blood clot. Based on our findings, UV- or NTAPPJ-treated titanium surfaces could significantly enhance the absorption of albumin, a major plasma protein, until 4 weeks after treatment. Albumin serves as a bridging scaffold to attract mesenchymal stem cells and promote their migration through its cell-attracting terminal Arg-Gly-Asp (RGD) sequence^12^. The ligand-binding interaction between the RGD terminal of the adsorbed protein and integrin from the cell membrane can act as a chemoattractant^47^. Thus, even if the hydrophilic state of the treated titanium surface cannot be maintained for 4 weeks needed for wound healing with marked woven bone formation and maturation after implantation, this state may promote the differentiation of already attached osteoblastic cells to the bone matrix formation/maturation and mineralization stages and maintain ALP activity^48^,^49^,^50^,^51^,^52^.

Titanium implant products, regardless of their applications in the medical and dental fields, are also commercially available as storage devices. Although UV and NTAPPJ can alleviate the biological aging of titanium until 4 weeks, i.e. at least the time required for initial healing, the requirement for treatment of the titanium surface for at least 10 min each time just before surgery may be challenging to surgeons in the operating room. Based on the results of our current study, pretreatment of titanium implants with UV or NTAPPJ may be applicable if appropriate storage methods are used to maintain the hydrophilicity of the titanium surface, similar to that observed immediately after treatment. Commercially, the ultimate goal is the development of treatment strategies using UV or NTAPPJ titanium implants that are not aged, regardless of the storage time.

Taken together, our findings demonstrated that UV and NTAPPJ treatment could improve the hydrophilicity of the titanium surface, contributing to enhancement and maintenance of the biological activity and interactions between blood proteins and osteoblastic cells until 4 weeks. These changes could thus increase early osseointegration between titanium implants and surrounding bone and reduce healing time, allowing patients to return to their normal lifestyles more quickly.

Several limitations to this study should be considered when interpreting these data. First, because UV is delivered to the entire titanium disc surface, NTAPPJ was focused on the titanium disc surface under jet plume, based on previous studies using the same method^15^,^16^,^29^,^53^. Previous studies have reported that NTAPPJ can affect the far side of a material from the irradiation centre owing to incorporation of air from the periphery of the jet or the admixture of a few percent with respect to the noble background gas^44^,^54^,^55^. Lee *et al.* reported that on the polystyrene plates, the range of the effects of NTAPPJ could be roughly calculated by the distance between the centre and far-side parts, at least 14.5 mm from the plume^55^. However, by LIF measurements, previous studies have revealed that the density of hydroxyl radicals is highest at the centre of the plasma jet on the metal surface, but decreases gradually as the distance from the axis increases^44^,^45^. Second, in this study, because the titanium samples were flat discs measuring 12 mm in diameter, the results from this study may not be applicable to the actual clinical setting, because the sizes of the titanium sample and the single plasma jet were too small to apply to complex prosthetic orthopaedic implants, particularly hip or knee joint prostheses in real situations. In order to facilitate processing of complex or large surfaces, other plasma sources, including multijet arrays^56^, spatially extended atmospheric plasma (SEAP) arrays^57^, and surface dielectric barrier discharge (DBD) plasma^58^, have been developed. Future studies using these sources need to determine the clinical applicability of nonthermal plasma in the treatment of complex and large orthopaedic implants. Additionally, *in vivo* experiments are necessary to confirm whether these preliminary results can be applied in real clinical situations in the medical and dental fields using inflammatory factor assays and to ensure the long-term survival rates of the titanium implants, particularly in compromised patients.

Despite the limitations of this study, we found that there were no differences in the effects of treatment using UV or NTAPPJ on the surfaces of grade IV machined titanium discs. Photocatalytic activity by UV and higher production of ROS or tentatively synergistic ROS/UV action by NTAPPJ altered the surface from negatively charged and hydrophobic (bioinert) to relatively positively charged and hydrophilic (bioactive), thereby enhancing protein adsorption, pre-osteoblastic cell attachment, and cytoskeleton development. Even if this effect may not last for 4 weeks to promote surrounding cell adhesion, the effects were sufficient to maintain ALP activity after 7 days of incubation. This positive effect of UV and NTAPPJ treatment could enhance the biological activity of titanium over time.

## Additional Information

**How to cite this article**: Choi, S.-H. *et al.* Time-dependent effects of ultraviolet and nonthermal atmospheric pressure plasma on the biological activity of titanium. *Sci. Rep.* **6**, 33421; doi: 10.1038/srep33421 (2016).
