# Corrigendum: Time-dependent effects of ultraviolet and nonthermal atmospheric pressure plasma on the biological activity of titanium

**DOI:** 10.1038/srep36430

**Published:** 2016-11-11

**Authors:** Sung-Hwan Choi, Won-Seok Jeong, Jung-Yul Cha, Jae-Hoon Lee, Hyung-Seog Yu, Eun-Ha Choi, Kwang-Mahn Kim, Chung-Ju Hwang

Scientific Reports 6: Article number: 3342110.1038/srep33421; published online: 09 15 2016; updated: 11 11 2016

This Article contains typographical errors. In the Materials and Methods section, under the subheading “Protein adsorption assay”

“The protein solution (100 μM; 1 mg/mL in phosphate-buffered saline [PBS], pH 7.4) was pipetted onto and spread over each sample surface immediately and 28 days after treatment using UV or NTAPPJ treatment”.

should read:

“The protein solution (100 μL; 1 mg/mL in phosphate-buffered saline [PBS], pH 7.4) was pipetted onto and spread over each sample surface immediately and 28 days after treatment using UV or NTAPPJ treatment”.

In the Results section, under the subheading “Changes in surface chemical compositions in the UV- and NTAPPJ-treated groups”

“However, the peaks of both UV- and NTAPJJ-treated groups were markedly decreased over time.”

Should read:

“However, the peaks of both UV- and NTAPPJ-treated groups were markedly decreased over time.”

